# The Impact of COVID-19 Pandemic Lockdown on the Relationship between Pediatric MAFLD and Renal Function

**DOI:** 10.3390/jcm12052037

**Published:** 2023-03-04

**Authors:** Maria Sole Valentino, Pierluigi Marzuillo, Claudia Esposito, Mario Bartiromo, Michele Nardolillo, Annalisa Valentina Villani, Alessandro Maresca, Giuseppe Furcolo, Stefano Guarino, Emanuele Miraglia del Giudice, Anna Di Sessa

**Affiliations:** 1 Department of Woman, Child, and General and Specialized Surgery, University of Campania “Luigi Vanvitelli”, 80138 Naples, Italy; 2 Unità Operativa Complessa di Pediatria e Pronto Soccorso Pediatrico, AORN Moscati, 83100 Avellino, Italy

**Keywords:** metabolic associated fatty liver disease, chronic kidney disease, renal function, COVID-19, lockdown, children

## Abstract

Background: Both direct and indirect effects of COVID-19 have been found in all age groups. In particular, adult data demonstrated significant changes in patients with chronic and metabolic disease (e.g., obesity, diabetes, chronic kidney disease (CKD), and metabolic associated fatty liver dysfunction (MAFLD)), while similar pediatric evidence is still limited. We aimed at investigating the impact of the COVID-19 pandemic lockdown on the relationship between MAFLD and renal function in children with CKD due to congenital abnormalities of the kidney and urinary tract (CAKUT). Methods: A total of 21 children with CAKUT and CKD ≥ stage 1 underwent a comprehensive evaluation within 3 months before and 6 months after the first Italian lockdown. Results: At follow-up, CKD patients with MAFLD presented higher BMI-SDS, serum uric acid, triglycerides, and microalbuminuria levels and lower eGFR levels than those without MAFLD (all *p* < 0.05). Higher ferritin and white blood cell concentrations were also found in patients with CKD diagnosed with MAFLD than peers without MAFLD (both *p* = 0.01). Compared to children without MAFLD, a higher delta of BMI-SDS, eGFR levels, and microalbuminuria levels was found in patients with MAFLD. Conclusions: Due to the negative influence of the COVID-19 lockdown on cardiometabolic health in childhood, a careful management of children with CKD is warranted.

## 1. Introduction

The ongoing coronavirus disease 19 [COVID-19] pandemic has disrupted lives across all countries [1,2], with a significant impact on morbidity and mortality [3,4]. As a result of the social distancing measurements to contain the spread of the infection, the subsequent lockdown has dramatically affected the daily routine of people worldwide [5,6]. Different medical and psychological effects have been reported both in adults and children who have experienced the lockdown [7,8,9,10].

More interestingly, a close relationship between COVID-19 and chronic diseases (including obesity, type 2 diabetes (T2D), and chronic liver, lung, and kidney disease) has been recently described both in adults [11,12,13] and in children [7,14,15] due to the bidirectional intimate link of COVID-19 and chronic diseases with inflammation [15,16]. In particular, liver abnormalities have been found to be significantly increased in COVID-19 patients [17,18], and a more severe infection has been reported in subjects with pre-existing liver diseases, such as metabolic associated fatty liver disease (MAFLD) [19,20,21]. This new nomenclature—primarily proposed for adults in 2020 [22] and then updated for children [23]—emphasized the close association of fatty liver with metabolic derangements by highlighting the cardiometabolic risk (e.g., metabolic syndrome, T2D, insulin resistance (IR), and cardiovascular disease) of these patients since childhood [24]. Recent adult data have demonstrated not only a close and independent association of MAFLD with chronic kidney disease (CKD) [13,25,26,27], but also its interplay with metabolic derangements [28,29], while evidence in childhood is still lacking.

Both conditions are chronic, progressive diseases with an increased global prevalence [30,31] sharing certain metabolic pathways (e.g., inflammation) [27,29,32,33]. From a pathophysiological point of view, MAFLD might contribute to CKD through several metabolic factors, including abdominal obesity, IR, inflammation, and oxidative stress [26,27,30].

In light of the impact of COVID-19 and lockdown measures on adult chronic diseases (e.g., MAFLD and CKD) [21,34] and the paucity of similar pediatric data, we aimed to investigate the influence of the COVID-19 pandemic lockdown on the relationship between MAFLD and renal function in childhood.

## 2. Materials and Methods

A total of 34 children and adolescents aged 5–17 years with congenital abnormalities of the kidney and urinary tract (CAKUT)-related CKD stages 1–4 consecutively attending our Nephrology Clinic for a regular follow-up were retrospectively selected. Patients observed within 3 months (from December 2019 to February 2020) before and within 6 months (from May 2020 to November 2020) after the end of the first Italian lockdown were retrospectively identified. Exclusion criteria were considered as: (i) denied consent to be included in the study or to undergo any procedure (*n* = 5); (ii) occurrence of urinary tract infection (UTI) (*n* = 3) and of COVID-19 infection (*n* = 5) between the two observation points. Therefore, we enrolled 21 patients. No patient dropouts occurred.

The Ethics Committee of our university approved the study (0010396/i). Written informed consent from all the children and their parents was obtained prior to commencing the study for diagnostic procedures and for anonymous processing of the data for retrospective studies.

Moreover, a historical cohort of 21 patients matched for age and sex, examined between 2004 and 2007 and who underwent two follow-up visits with an interval time between the two visits of 11–14 months, was also selected.

Anthropometric, laboratory, and instrumental data were retrospectively collected from the clinical charts between December 2021 and May 2022.

At every follow-up visit (before and after lockdown), all the enrolled subjects routinely underwent a comprehensive clinical, biochemical, urinary, and instrumental assessment. In our clinical practice, these evaluations are commonly included in the regular follow-up to ensure an accurate monitoring of renal function in such at-risk patients.

Physical examinations were conducted by trained physicians. Anthropometric measurements were obtained in a standing position while lightly clothed and without shoes, as described elsewhere [24]. 

Blood pressure was obtained by three oscillometric measurements taken with an appropriate-sized arm cuff every five minutes, and high blood pressure levels were confirmed by using the auscultatory method (taking three additional measurements). Blood samples were collected after an overnight fast [24].

Daily proteinuria was measured as described elsewhere [35]. Albuminuria was defined as the presence of the albuminuria creatinuria ratio of ACR ≥ 30 mg/g. Normal urinary albumin excretion was defined as an ACR of <30 mg/g. Microalbuminuria was defined as an ACR ranging from 30 to 300 mg/g. 

Serum creatinine levels were estimated through the Jaffe method, as traditionally used in our laboratories. Due to the absence of the modified assay for creatinine, the eGFR was calculated by using the original Schwartz equation and then normalized to the ideal body weight-derived body surface area [36].

MAFLD diagnosis was based on the radiological evidence of hepatic steatosis and the presence of at least one of the following criteria, namely, overweight/obesity, T2D, or evidence of metabolic dysregulation (defined as the presence of two or more of these conditions: (1) Waist circumference > 95th percentile for age and sex; (2) Blood pressure > 95th for age, sex, and height; (3) Triglycerides > 150 mg/dL; (4) HDL < 40 mg/dl; (5) prediabetes; (6) homeostasis model assessment-insulin resistance (HOMA-IR) score > 2.5; and (7) C-reactive protein (CRP) levels > 2 mg/L) [22,23]. 

### Statistical Analysis

Data were expressed as mean ± SD or proportions (%). We classified the study population according to MAFLD presence/absence, and we compared the main features between these groups at baseline and at follow-up observations. The delta between both observations (pre- and post- lockdown) was also calculated for the main characteristics, and differences in these values in patients with and without MAFLD were examined.

Differences for continuous variables were analyzed with the independent sample t-test for normally distributed variables and with the Mann–Whitney test in case of non-normality. Qualitative variables were compared by using the Chi-square or Fisher’ exact test, as appropriate. Significance was considered at the level of *p* < 0.05.

The IBM SPSS Statistics software, Version 24 (IBM, Armonk, NY, USA), was used for all statistical analyses.

## 3. Results

The mean age of the study population was 10.63 ± 4.8 years. The main characteristics at baseline (pre-lockdown) and at follow-up (post-lockdown) of patients stratified according to pediatric MAFLD definition are shown in Table 1. At baseline, CKD subjects with MAFLD did not show significant differences for the main anthropometric variables (Table 1). Baseline serum triglycerides levels were significantly higher in CKD patients with MAFLD than in those without MAFLD (*p* = 0.04). At follow-up, CKD patients with MAFLD showed higher BMI-SDS and DBP-SDS values than those without MAFLD (both *p* = 0.03). Moreover, increased serum uric acid, triglycerides, and microalbuminuria levels and lower eGFR levels were found in children with MAFLD compared to those without MAFLD (*p* = 0.01, *p* = 0.03, *p* = 0.04, and *p* = 0.03, respectively) (Table 1). As inflammation markers, higher ferritin and white blood cell concentrations were also found in patients with CKD diagnosed with MAFLD than peers without MAFLD (both *p* = 0.01). 

Compared to patients without MAFLD, children with MAFLD showed—between the two observation points—a significantly higher delta of BMI-SDS (0.52 ± 1.23 vs. 0.44 ± 0.49, *p* = 0.01), of eGFR levels (11.65 ± 15.95 vs. 4.33 ± 7.01, *p* = 0.02), and of microalbuminuria levels (122.01 ± 129.45 vs. 25.33 ± 33.16, *p* = 0.04).

In the historical cohort, the mean age (10.8 ± 5.1 SDS) was similar to that of the examined cohort for this research (*p* = 0.77). Compared to the present cohort, in the historical group, 6/21 patients (28.5%) were diagnosed with MAFLD (*p* = 0.69), and a significantly lower increase of MAFLD parameters was observed (delta of BMI-SDS 0.19 ± 0.38, delta of DBP-SDS 0.34 ± 0.62, and delta of Triglycerides 32.21 ± 20.75) compared to those observed in the present cohort (*p* for delta BMI-SDS 0.02, for DBP-SDS 0.02, and for Triglycerides 0.03, respectively).

## 4. Discussion

Our findings showed for the first time a negative impact of the COVID-19 lockdown on renal function in a pediatric cohort with MAFLD and CKD. Due to certain shared pathogenic factors, including inflammation and IR, metabolic derangements represent common features both in MAFLD and CKD patients. Several studies have examined the relationship of chronic diseases with COVID-19 infection [12,13,15,19], but to our knowledge, no current pediatric evidence is available on the consequences of the coexistence of such conditions during lockdown. 

In light of the role of obesity as a potential risk factor for SARS-CoV-2 infection and a severe COVID-19 clinical course [37,38,39], the association of COVID-19 with MAFLD has received increasing scientific attention in adult populations, but evidence in childhood is still limited [39,40].

To complicate matters, the role of obesity as a risk factor for severe COVID-19 illness has been demonstrated [37,38,41]. In particular, an increased risk of severe COVID-19 illness more than sixfold has been found for adult patients with MAFLD [38]. Of interest, this has been also demonstrated in young patients with MAFLD [40,42]. 

From this perspective, prevention of SARS-CoV-2 infection and weight control in pediatric MAFLD patients (as children at higher intrinsic cardiometabolic risk) represent crucial steps in this sensitive period of life [43].

Nevertheless, our observations contributed to add and expand current knowledge of the impact of the COVID-19 lockdown on the renal health of children with preexisting chronic diseases, such as MAFLD and CKD. Based on our preliminary but intriguing data, we speculate that the adverse effect of the COVID-19 lockdown on kidney function in children with MAFLD and CKD might be due to a vicious circle realized by the shared pathophysiological factors (e.g., inflammation and IR) and by certain environmental determinants, including increased physical inactivity and sedentary behaviors, related to the lockdown [39]. In fact, lifestyle interventions, including weight loss and regular physical activity, play a key role not only for MAFLD treatment [43], but also for the overall management of patients with CAKUT-related renal impairment [35]. As previously demonstrated [44], during the lockdown, a significant percentage of children with CKD showed not only disease progression, but also an increased BMI mean, likely due to less adherence to the Mediterranean diet and increased negative eating habits. Given also the intricate pathophysiological link between microalbuminuria and IR [45,46], the dysmetabolism that occurred in these children during lockdown [44,47] might explain both the large change in albuminuria and the potentially higher MAFLD risk in these patients.

Besides the well-known direct impact of COVID-19 on people’s health [19,39,48], several indirect effects of the related lockdown measures have been also reported [48,49,50,51]. In particular, metabolic health seemed to be dramatically affected by the lifestyle changes that occurred during lockdown [40,49,50,51,52], resulting in a significant worsening of the spectrum of metabolic abnormalities (e.g., body composition, fatty liver, metabolic syndrome, and glucose homeostasis), both in adults and children [51,52,53,54]. As childhood has been regarded as the most delicate and crucial period of life, with a greater vulnerability to the effects of cardiometabolic risk [55], the long-term consequences of the impact of the lockdown measures need to be adequately addressed.

However, this research has some limitations that need to be mentioned. The design of our preliminary study was retrospective, and the number of enrolled subjects—although well-phenotyped at both observation points—was limited. Both aspects prevent the generalizability of our results, but we were able to draw findings of clinical relevance. Moreover, data regarding WC, CRP, and HOMA-IR values were available only in a non-significant group of patients. Hepatic steatosis was diagnosed by liver ultrasound—as commonly used in clinical practice—instead of by biopsy [56]. Indeed, although this latter is still considered as the gold standard for the diagnosis of the disease, it represents an invasive procedure with ethical concerns, and its use has been approved in selected cases in childhood [56].

In view of the dangerous liaison of MAFLD with CKD [13,39] and of the putative effect of the COVID-19 outbreak lockdown on this association, a careful management of these patients should be warranted to prevent the serious cardiometabolic burden of these intertwined diseases.

## Figures and Tables

**Table 1:** Main features of the patients with CKD at baseline and at follow-up.

	Patients with CKD at Baseline			Patients with CKD at Follow-Up		
	No MAFLD (*n* = 14)	MAFLD (*n* = 7)	*p*-Value	No MAFLD (*n* = 14)	MAFLD (*n* = 7)	*p*-Value
**BMI-SDS**	0.04 ± 1.06	0.25 ± 1.87	0.86	0.29 ± 1.08	1.17 ± 1.32	**0.03**
**Sex (male), No. (%)**	8 (57.1)	3 (42.8)	0.81	9 (64.2%)	4 (57.1%)	0.68
**Stage 1 CKD, No. (%)**	5 (35.7)	2 (28.5)	0.92	4 (28.5)	1 (14.2)	0.77
**Stage 2 CKD, No. (%)**	9 (64.2)	3 (42.9)	0.87	10 (71.4)	3 (42.8)	0.49
**Stage 3 CKD, No. (%)**	0 (0)	1 (14.3)	0.99	0 (0)	2 (28.5%)	0.99
**Stage 4 CKD, No. (%)**	0 (0)	1 (14.3)	0.99	0 (0)	1 (14.3)	0.99
**Stage 5 CKD, No (%)**	0 (0)	0 (0)	0.99	0 (0)	0 (0)	0.99
**SBP-SDS**	0.09 ± 1.17	1.04 ± 1.20	0.51	0.92 ± 1.08	0.93 ± 0.93	0.96
**DBP-SDS**	0.07 ± 0.54	0.24 ± 0.50	0.23	0.18 ± 0.75	1.03 ± 1.15	**0.03**
**ALT, U/L, U/L**	18.91 ± 7.19	19.20 ± 4.38	0.76	21.84 ± 6.84	21.56 ± 8.32	0.87
**AST, U/L**	20.61 ± 6.1121	23 ± 5.05	0.62	23.45 ± 4.96	24.20 ± 7.05	0.12
**Phosphorus, mg/dL**	4.10 ± 0.62	4.36 ± 1.38	0.80	154.44 ± 34.08	162.63 ± 26.85	0.25
**Total-Cholesterol, mg/dL**	159.18 ± 14.93	172.00 ± 21.32	0.34	158.58 ± 14.39	174.01 ± 20.12	0.25
**Triglycerides, mg/dL**	91.76 ± 45.34	98.33 ± 42.18	**0.04**	78.01 ± 20.50	157.50 ± 15.78	**0.03**
**Glycemia, mg/dL**	76.89 ± 8.83	81.03 ± 7.55	0.87	77.31 ± 5.91	82.11 ± 5.43	0.09
**Uric acid, mg/dl**	5.18 ± 1.62	5.48 ± 1.17	0.73	5.50 ± 1.02	7.38 ± 0.84	**0.01**
**Hemoglobin, g/dl**	13.44 ± 1.51	14.38 ± 1.31	0.12	13.33 ± 1.49	14.22 ± 1.31	0.16
**White Blood Cells, ×10^3^/µL**	6988.56 ± 3702.49	7412.42 ± 1177.96	0.74	6813.33 ± 936.52	9457.50 ± 2935.17	**0.01**
**eGFR, mL/min/1.73 m^2^**	92.08 ± 9.02	85.95 ± 17.55	0.33	91.55 ± 10.88	76.53 ± 17.22	**0.03**
**Neutrophils, ×10^3^/µL**	4037.50 ± 1487.39	3226.00 ± 921.07	0.28	3815.83 ± 880.77	5200.01 ± 1974.84	0.06
**Platelets, ×10^3^/µL**	251,636.36 ± 76,322.04	278,750.01 ± 101,032.58	0.68	25,625.00 ± 74,504.57	278,761.00 ± 101,042.65	0.63
**Urea, mg/dl**	48.92 ± 12.46	44.60 ± 15.59	0.55	45.92 ± 11.62	53.17 ± 15.96	0.28
**Ferritin, µg/L**	21.01 ± 4.24	30.01 ± 5.75	0.44	12.75 ± 10.46	40.67 ± 9.61	**0.01**
**Parathormone, pg/mL**	21.51 ± 9.42	13.83 ± 8.27	0.25	26.11 ± 6.12	17.42 ± 13.06	0.12
**Vitamin D, ng/mL**	29.20 ± 13.89	16.10 ± 1.97	0.27	26.43 ± 9.07	27.56 ± 16.55	0.87
**Microalbuminuria, mg/L**	47.30 ± 90.87	207.60 ± 411.31	0.24	115.40 ± 145.28	374.67 ± 559.71	**0.04**
**UPr/UCr, mg/mg**	0.27 ± 0.35	0.54 ± 0.81	0.40	0.31 ± 0.33	1.01 ± 1.27	0.09

Abbreviations: ALT, alanine transaminase; AST, aspartate transaminase; BMI, body mass index;CKD, chronic kidney disease; DBP, diastolic blood pressure; eGFR, estimated glomerular filtration rate; MAFLD, Metabolic associated fatty liver disease; SBP, systolic blood pressure; SDS, standard deviation score; UPr/UCr, urinary protein/creatinine ratio. Bold values are for statistical significance.

## Data Availability

The data presented in this study are available on request from the corresponding author. The data are not publicly available due to the presence of information that could compromise research participant privacy.

## References

[1] Zhu N., Zhang D., Wang W., Li X., Yang B., Song J., Zhao X., Huang B., Shi W., Lu R. (2020). A Novel Coronavirus from Patients with Pneumonia in China, 2019. N. Engl. J. Med..

[2] Acuti Martellucci C., Flacco M.E., Cappadona R., Bravi F., Mantovani L., Manzoli L. (2020). SARS-CoV-2 pandemic: An overview. Adv. Biol. Regul..

[3] Chang D., Chang X., He Y., Tan K.J.K. (2022). The determinants of COVID-19 morbidity and mortality across countries. Sci. Rep..

[4] McGowan V.J., Bambra C. (2022). COVID-19 mortality and deprivation: Pandemic, syndemic, and endemic health inequalities. Lancet Public Health.

[5] Haucke M., Heinz A., Liu S., Heinzel S. (2022). The Impact of COVID-19 Lockdown on Daily Activities, Cognitions, and Stress in a Lonely and Distressed Population: Temporal Dynamic Network Analysis. J. Med. Internet Res..

[6] de Palma A., Vosough S., Liao F. (2022). An overview of effects of COVID-19 on mobility and lifestyle: 18 months since the outbreak. Transp. Res. Part A Policy Pract..

[7] Rundle A.G., Park Y., Herbstman J.B., Kinsey E.W., Wang Y.C. (2020). COVID-19-Related School Closings and Risk of Weight Gain Among Children. Obesity.

[8] Fegert J.M., Vitiello B., Plener P.L., Clemens V. (2020). Challenges and burden of the Coronavirus 2019 (COVID-19) pandemic for child and adolescent mental health: A narrative review to highlight clinical and research needs in the acute phase and the long return to normality. Child Adol. Psychiatry Men. Health.

[9] Pierce M., Hope H., Ford T., Hatch S., Hotopf M., John A., Kontopantelis E., Webb R., Wessely S., McManus S. (2020). Mental health before and during the COVID-19 pandemic: A longitudinal probability sample survey of the UK population. Lancet Psychiatry.

[10] Giuntella O., Hyde K., Saccardo S., Sadoff S. (2021). Lifestyle and mental health disruptions during COVID-19. Proc. Natl. Acad. Sci. USA.

[11] Targher G., Mantovani A., Wang X.B., Yan H.D., Sun Q.F., Pan K.H., Byrne C.D., Zheng K.I., Chen Y.P., Eslam M. (2020). Patients with diabetes are at higher risk for severe illness from COVID-19. Diabetes Metab..

[12] Marjot T., Moon A.M., Cook J.A., Abd-Elsalam S., Aloman C., Armstrong M.J., Pose E., Brenner E.J., Cargill T., Catana M.A. (2021). Outcomes following SARS-CoV-2 infection in patients with chronic liver disease: An international registry study. J. Hepatol..

[13] Liang Y., Chen H., Liu Y., Hou X., Wei L., Bao Y., Yang C., Zong G., Wu J., Jia W. (2022). Association of MAFLD With Diabetes, Chronic Kidney Disease, and Cardiovascular Disease: A 4.6-Year Cohort Study in China. J. Clin. Endocrinol. Metab..

[14] Di Sessa A., Lanzaro F., Zarrilli S., Picone V., Guarino S., Miraglia Del Giudice E., Marzuillo P. (2021). COVID-19 and pediatric fatty liver disease: Is there interplay?. World J. Gastroenterol..

[15] Moore J.B. (2022). COVID-19, childhood obesity, and NAFLD: Colliding pandemics. Lancet Gastroenterol. Hepatol..

[16] Sharma P., Kumar A., Anikhindi S., Bansal N., Singla V., Shivam K., Arora A. (2021). Effect of COVID-19 on Pre-existing Liver disease: What Hepatologist Should Know?. J. Clin. Exp. Hepatol..

[17] Tellez L., Martin Mateos R.M. (2020). COVID-19 and liver disease: An update. Gastroenterol. Hepatol..

[18] Luo M., Ballester M.P., Soffientini U., Jalan R., Mehta G. (2022). SARS-CoV-2 infection and liver involvement. Hepatol. Int..

[19] Targher G., Mantovani A., Byrne C.D., Wang X.B., Yan H.D., Sun Q.F., Pan K.H., Zheng K.I., Chen Y.P., Eslam M. (2020). Risk of severe illness from COVID-19 in patients with metabolic dysfunction-associated fatty liver disease and increased fibrosis scores. Gut.

[20] Chen H., Chen Q. (2022). COVID-19 Pandemic: Insights into Interactions between SARS-CoV-2 Infection and MAFLD. Int. J. Biol. Sci..

[21] Xu Y., Yang X., Bian H., Xia M. (2021). Metabolic dysfunction associated fatty liver disease and coronavirus disease 2019: Clinical relationship and current management. Lipids Health Dis..

[22] Eslam M., Sanyal A.J., George J., International Consensus P. (2020). MAFLD: A Consensus-Driven Proposed Nomenclature for Metabolic Associated Fatty Liver Disease. Gastroenterology.

[23] Eslam M., Newsome P.N., Sarin S.K., Anstee Q.M., Targher G., Romero-Gomez M., Zelber-Sagi S., Wong V.W.S., Dufour J.F., Schattenberg J.M. (2020). A new definition for metabolic dysfunction-associated fatty liver disease: An international expert consensus statement. J. Hepatol..

[24] Di Bonito P., Valerio G., Licenziati M.R., Campana G., Del Giudice E.M., Di Sessa A., Morandi A., Maffeis C., Chiesa C., Pacifico L. (2021). Uric acid, impaired fasting glucose and impaired glucose tolerance in youth with overweight and obesity. Nutr. Metab. Cardiovasc. Dis..

[25] Sun D.Q., Jin Y., Wang T.Y., Zheng K.I., Rios R.S., Zhang H.Y., Targher G., Byrne C.D., Yuan W.J., Zheng M.H. (2021). MAFLD and risk of CKD. Metabolism.

[26] Hu Q., Chen Y., Bao T., Huang Y. (2022). Association of metabolic dysfunction-associated fatty liver disease with chronic kidney disease: A Chinese population-based study. Ren. Fail..

[27] Wang T.Y., Wang R.F., Bu Z.Y., Targher G., Byrne C.D., Sun D.Q., Zheng M.H. (2022). Association of metabolic dysfunction-associated fatty liver disease with kidney disease. Nat. Rev. Nephrol..

[28] Su W., Chen M., Xiao L., Du S., Xue L., Feng R., Ye W. (2022). Association of metabolic dysfunction-associated fatty liver disease, type 2 diabetes mellitus, and metabolic goal achievement with risk of chronic kidney disease. Front. Public Health.

[29] Theofilis P., Vordoni A., Kalaitzidis R.G. (2022). Interplay between metabolic dysfunction-associated fatty liver disease and chronic kidney disease: Epidemiology, pathophysiologic mechanisms, and treatment considerations. World J. Gastroenterol..

[30] Hashimoto Y., Hamaguchi M., Okamura T., Nakanishi N., Obora A., Kojima T., Fukui M. (2022). Metabolic associated fatty liver disease is a risk factor for chronic kidney disease. J. Diabetes. Investig..

[31] Eslam M., El-Serag H.B., Francque S., Sarin S.K., Wei L., Bugianesi E., George J. (2022). Metabolic (dysfunction)-associated fatty liver disease in individuals of normal weight. Nat. Rev. Gastroenterol. Hepatol..

[32] Kaya E., Yilmaz Y. (2022). Metabolic-associated Fatty Liver Disease (MAFLD): A Multi-systemic Disease Beyond the Liver. J. Clin. Transl. Hepatol..

[33] Deng Y., Zhao Q., Gong R. (2021). Association Between Metabolic Associated Fatty Liver Disease and Chronic Kidney Disease: A Cross-Sectional Study from NHANES 2017–2018. Diabetes Metab. Syndr. Obes..

[34] Tao Z., Li Y., Cheng B., Zhou T., Gao Y. (2021). Risk of Severe COVID-19 Increased by Metabolic Dysfunction-associated Fatty Liver Disease: A Meta-analysis. J. Clin. Gastroenterol..

[35] Marzuillo P., Guarino S., Di Sessa A., Rambaldi P.F., Reginelli A., Vacca G., Cappabianca S., Capalbo D., Esposito T., De Luca Picione C. (2021). Congenital Solitary Kidney from Birth to Adulthood. J. Urol..

[36] La Scola C., Guarino S., Pasini A., Capalbo D., Liguori L., Di Sessa A., Bertulli C., Mencarelli F., De Mutiis C., Campana G. (2020). Effect of Body Mass Index on Estimated Glomerular Filtration Rate Levels in Children With Congenital Solitary Kidney: A Cross-Sectional Multicenter Study. J. Ren. Nutr..

[37] Liu D., Zhang T., Wang Y., Xia L. (2021). The Centrality of Obesity in the Course of Severe COVID-19. Front. Endocrinol..

[38] Zheng K.I., Gao F., Wang X.B., Sun Q.F., Pan K.H., Wang T.Y., Ma H.L., Chen Y.P., Liu W.Y., George J. (2020). Letter to the Editor: Obesity as a risk factor for greater severity of COVID-19 in patients with metabolic associated fatty liver disease. Metabolism.

[39] Stefan N., Birkenfeld A.L., Schulze M.B. (2021). Global pandemics interconnected -obesity, impaired metabolic health and COVID-19. Nat. Rev. Endocrinol..

[40] Zhou Y.J., Zheng K.I., Wang X.B., Yan H.D., Sun Q.F., Pan K.H., Wang T.Y., Ma H.L., Chen Y.P., George J. (2020). Younger patients with MAFLD are at increased risk of severe COVID-19 illness: A multicenter preliminary analysis. J. Hepatol..

[41] Gao F., Zheng K.I., Wang X.B., Sun Q.F., Pan K.H., Wang T.Y., Chen Y.P., Targher G., Byrne C.D., George J. (2020). Obesity Is a Risk Factor for Greater COVID-19 Severity. Diabetes Care.

[42] Ji D., Qin E., Lau G. (2020). Reply to: ‘Younger patients with MAFLD are at increased risk of severe COVID-19 illness: A multicenter preliminary analysis’. J. Hepatol..

[43] Zhou Y.H., Rios R.S., Zheng K.I., Zheng M.H. (2021). Recommendations and Clinical Guidance for Children with Metabolic-associated Fatty Liver Disease during the COVID-19 Pandemic. J. Clin. Transl. Hepatol..

[44] Palma P.L., Sessa A.D., Passaro A.P., Palladino E., Furcolo G., Barlabà A., Rivetti G., Lucia M., Miraglia Del Giudice E., Guarino S. (2023). Effects of Lockdown for COVID-19 Pandemic on Chronic Kidney Disease Progression in Children with Congenital Anomalies of the Kidney and Urinary Tract: A Retrospective Pilot Study. Children.

[45] Colasante A.M., Bartiromo M., Nardolillo M., Guarino S., Marzuillo P., Mangoni di SStefano G.S.R.C., Miraglia Del Giudice E., Di Sessa A. (2022). Tangled relationship between insulin resistance and microalbuminuria in children with obesity. World J. Clin. Pediatr..

[46] Gu S., Wang A., Ning G., Zhang L., Mu Y. (2020). Insulin resistance is associated with urinary albumin-creatinine ratio in normal weight individuals with hypertension and diabetes: The REACTION study. J. Diabetes.

[47] Capra M.E., Stanyevic B., Giudice A., Monopoli D., Decarolis N.M., Esposito S., Biasucci G. (2022). The Effects of COVID-19 Pandemic and Lockdown on Pediatric Nutritional and Metabolic Diseases: A Narrative Review. Nutrients.

[48] Madjid M., Safavi-Naeini P., Solomon S.D., Vardeny O. (2020). Potential Effects of Coronaviruses on the Cardiovascular System: A Review. JAMA Cardiol..

[49] Auriemma R.S., Pirchio R., Liccardi A., Scairati R., Del Vecchio G., Pivonello R., Colao A. (2021). Metabolic syndrome in the era of COVID-19 outbreak: Impact of lockdown on cardiometabolic health. J. Endocrinol. Invest..

[50] Bérard E., Huo Yung Kai S., Coley N., Bongard V., Ferrières J. (2022). One-Year Impact of COVID-19 Lockdown-Related Factors on Cardiovascular Risk and Mental Health: A Population-Based Cohort Study. Int. J. Environ. Res. Public Health.

[51] López-González Á.A., Altisench Jané B., Masmiquel Comas L., Arroyo Bote S., González San Miguel H.M., Ramírez Manent J.I. (2022). Impact of COVID-19 Lockdown on Non-Alcoholic Fatty Liver Disease and Insulin Resistance in Adults: A before and after Pandemic Lockdown Longitudinal Study. Nutrients.

[52] Ojo O., Wang X.H., Ojo O.O., Orjih E., Pavithran N., Adegboye A.R.A., Feng Q.Q., McCrone P. (2022). The Effects of COVID-19 Lockdown on Glycaemic Control and Lipid Profile in Patients with Type 2 Diabetes: A Systematic Review and Meta-Analysis. Int. J. Environ. Res. Public Health.

[53] Welling M.S., Abawi O., van den Eynde E., van Rossum E.F.C., Halberstadt J., Brandsma A.E., Kleinendorst L., van den Akker E.L.T., van der Voorn B. (2022). Impact of the COVID-19 Pandemic and Related Lockdown Measures on Lifestyle Behaviors and Well-Being in Children and Adolescents with Severe Obesity. Obes. Facts..

[54] Sforza C. (2022). COVID-19 Lockdown, Sedentarism, Metabolic Alterations, Obesity: Can We Reverse the Domino Effect in Children?. Children.

[55] Drozdz D., Alvarez-Pitti J., Wójcik M., Borghi C., Gabbianelli R., Mazur A., Herceg-Čavrak V., Lopez-Valcarcel B.G., Brzeziński M., Lurbe E. (2021). Obesity and Cardiometabolic Risk Factors: From Childhood to Adulthood. Nutrients.

[56] Marzuillo P., Grandone A., Perrone L., Miraglia Del Giudice E. (2015). Controversy in the diagnosis of pediatric non-alcoholic fatty liver disease. World J. Gastroenterol..

